# A missed case of intraductal oncocytic papillary neoplasm associated with missed stones in extrahepatic bile duct: a case report

**DOI:** 10.3389/fonc.2024.1349914

**Published:** 2024-05-21

**Authors:** Cong Xie, Hang Zhang, Yushan Meng, Bin Cao

**Affiliations:** ^1^ Department of Gastroenterology, The Affiliated Hospital of Qingdao University, Qingdao, China; ^2^ Department of Medicine, Qingdao University, Qingdao, China

**Keywords:** carcinoma of the bile duct, intraductal oncocytic papillary neoplasm of the bile duct, gallstones, missed diagnosis, choledochoscopy

## Abstract

The pathological features of intraductal oncocytic papillary neoplasm (IOPN) of the bile duct include tumor cells that are rich in eosinophilic cytoplasm and arranged in papillary structures. Herein, we report a missed case of IOPN of the bile duct because of concomitant gallstones. A 70-year-old woman was hospitalized with upper abdominal discomfort. The primary diagnosis was choledocholithiasis following imaging examination. However, an unidentified mass was detected after the gallstones were removed. The mass appeared as many papillary protuberances surrounded by fish-egg-like mucosa when viewed by the choledochoscope and was confirmed as IOPN by pathological examination. The patient underwent choledochectomy and no recurrence was observed at the 6-month follow-up examination. In this report, peroral choledochoscopy demonstrated its advantages for the diagnosis of biliary diseases and acquisition of tissue specimens. Therefore, it may solve the challenge related to the lack of preoperative pathological evidence for bile duct tumors.

## 1 Introduction

Carcinomas of the extrahepatic bile duct are a heterogeneous group of cancers, which are often diagnosed at an advanced stage and exhibit poor patient outcomes. Based on “*WHO Classification of Tumours: Digestive System Tumours (5^th^ Edition)*”, it can be classified into cholangiocarcinoma, intraductal papillary neoplasm of the bile duct (IPNB), squamous cell carcinoma, adenosquamous carcinoma, and undifferentiated carcinoma. Moreover, IPNB can be further divided into pancreatobiliary, gastric, intestinal, and oncocytic type (1–3). Among these classifications, intraductal oncocytic papillary neoplasm (IOPN) of the bile duct is extremely rare. Only 20 cases with complete information were retrieved from the PubMed database, including only five patients with neoplasm in the extrahepatic bile duct. We encountered a rare case of a patient with IOPN of the extrahepatic bile duct. The novelty of this case is the co-existence of tumor and gallstones, which contributed to the missed diagnosis and is reported here for the first time. This case is also the first report to reveal the choledochoscopic findings of IOPN of the bile duct.

## 2 Manuscript

### 2.1 Case report

**Figure 1:**
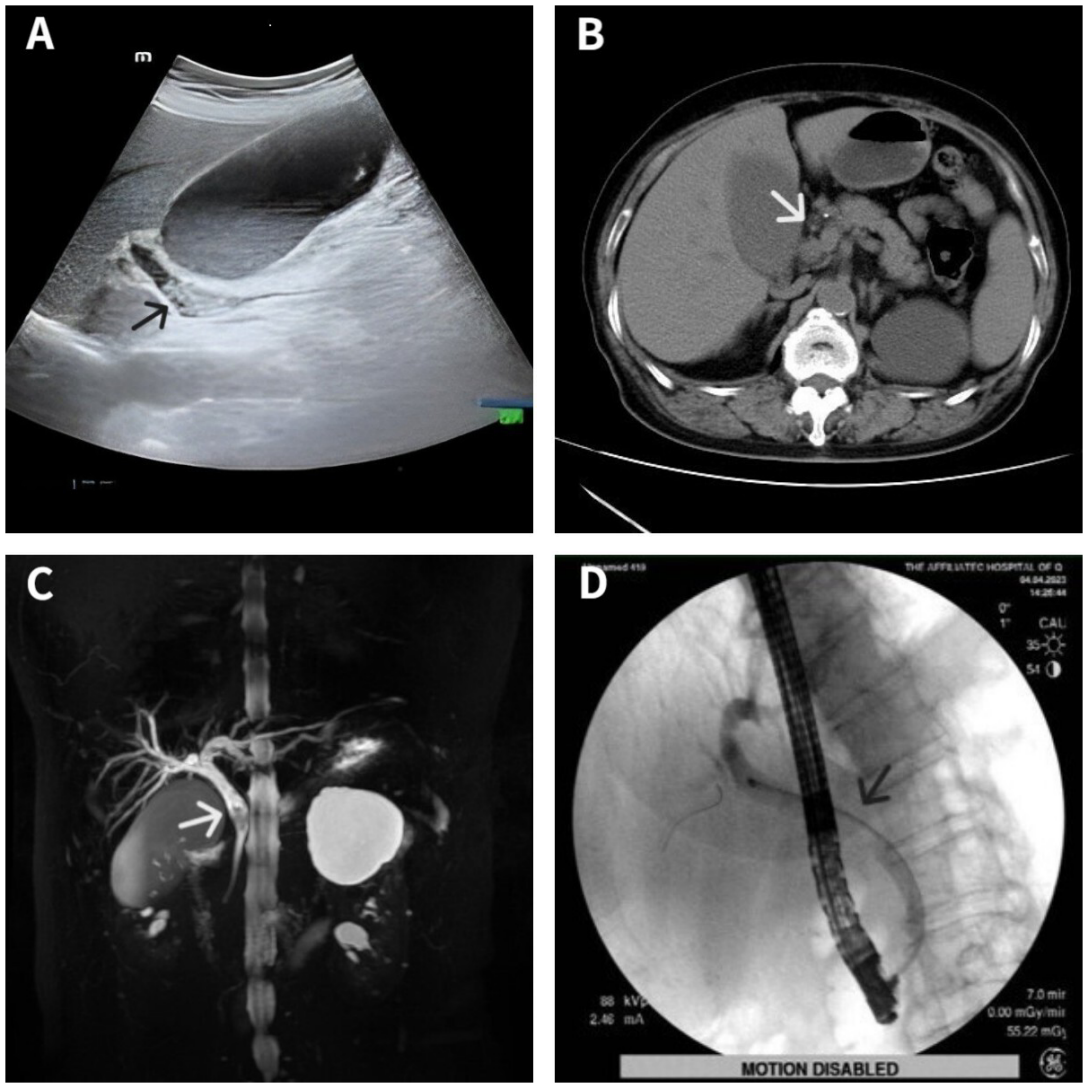
Imaging data of the patient. Ultrasonography showed several hyperechoic masses within the dilated bile duct **(A)**. Computed tomography showed some slightly high-density masses in the bile duct **(B)**. Magnetic resonance cholangiopancreatography showed some hypointense masses within the dilated bile duct **(C)**. Endoscopic retrograde cholangiopancreatography showed a filling defect at the middle bile duct after several stones were removed **(D)**. Lesion locations are marked with arrows.

A 70-year-old woman was hospitalized with upper abdominal discomfort. The patient had a history of carbon monoxide poisoning and was diagnosed with diabetes and hypertension. The physical examination findings were unremarkable. Laboratory tests showed above normal levels of total bilirubin ↑ (81.5 umo1/L; normal value: 3-22 umo1/L), direct bilirubin ↑ (42.9 umo1/L; 0-8 umo1/L), alanine aminotransferase ↑ (330.4 U/L; 7-40 U/L), aspartate aminotransferase ↑ (99.7 U/L; 13-35 U/L), γ-glutamyl transferase ↑ (464.0 U/L; 7-45 U/L), alkaline phosphatase ↑ (138.8 U/L; 50-135 U/L), and C-reactive protein ↑ (5.08 mg/L; 0-5 mg/L). Conversely, the carbohydrate antigen 19-9 (CA19-9) level, carcinoembryonic antigen (CEA) level, and leukocyte count were all normal. Ultrasonography showed a dilated bile duct with a diameter of 1.2 cm. Several hyperechoic masses with a diameter of 0.4-0.8 cm were observed in the bile duct, which exhibited apparent acoustic shadows (**Figure 1A**). Computed tomography (CT) also revealed multiple slightly high-density masses within the dilated bile duct (**Figure 1B**). Magnetic resonance cholangiopancreatography (MRCP) demonstrated hypointense masses in the bile duct (**Figure 1C**).

**Figure 2:**
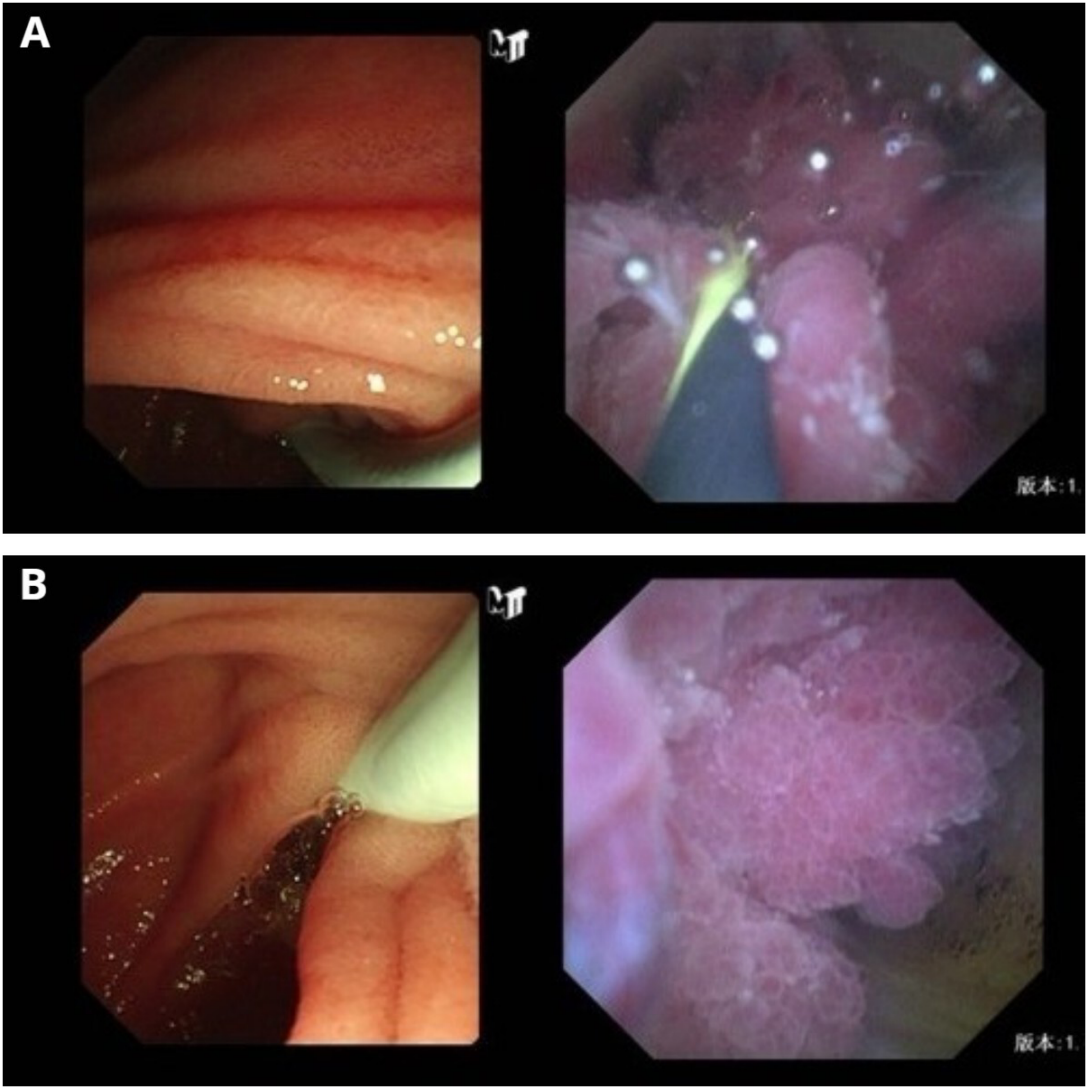
Choledochoscopic findings of intraductal oncocytic papillary neoplasm of the bile duct. Choledochoscopy showed many papillary protuberances **(A)** surrounded by fish-egg-like mucosa **(B)**.

**Figure 3:**
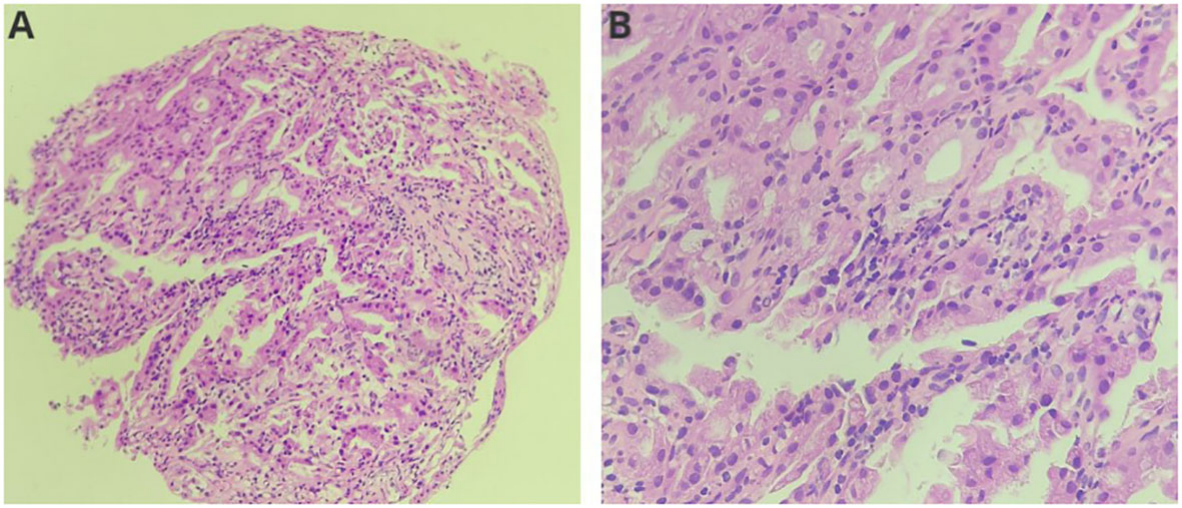
Histological features of the neoplasm (**(A)**: HE, ×200; **(B)**: HE, ×400). The neoplasm showed papillary structures with fibrovascular cores. Neoplasm cells contained a large amount of eosinophilic cytoplasm and round nuclei.

The diagnosis was choledocholithiasis based on the above evidence. Therefore, the patient underwent endoscopic retrograde cholangiopancreatography (ERCP). Several stones were removed during the operation. However, a filling defect was still observed at the middle bile duct (**Figure 1D**). Five days after the stones were removed by ERCP, the patient underwent peroral choledochoscopy. Peroral choledochoscopy (SpyGlass DS II, Boston Scientific Corporation, Delaware, United States) showed that the mass appeared as many papillary protuberances surrounded by fish-egg-like mucosa (**Figure 2**). The purplish-red lesion extended over half of the circumference of the bile duct. We performed the biopsy under direct vision of the choledochoscope, and the pathological diagnosis was IOPN. The histological appearance was papillary structures with fibrovascular cores, and the tumor cells contained a large amount of eosinophilic cytoplasm and possessed round nuclei. Additionally, cell atypia was light to moderate and local glands were hyperplastic in a crowded state (**Figure 3**). Finally, no distant metastasis or involvement of regional lymph nodes was apparent. Eleven days after the biopsy under choledochoscopy was performed, the patient underwent choledochectomy. We removed the common bile duct and performed Roux-en-Y choledochojejunostomy. Although, postoperative adjuvant chemotherapy was not performed, no recurrence was detected at 6 months after surgery.

### 2.2 Literature survey

We found 20 cases with complete data through October 2023 in the PubMed database using “intraductal oncocytic papillary neoplasm of the bile duct” as the search query (4–16). The information related to this search is presented in **Table 1**. Overall, 12 (60%) patients were from Japan and six (30%) were from the USA. The mean age was 57.2 years (minimum value: 38 years; maximum value: 71 years) and only six (30%) patients were female. Patients had different clinical symptoms, and the most common presentation was abdominal pain. In addition, some patients were found incidentally during health examinations or tests for other diseases.

**Table 1:** Basic clinical data of reported cases.

No.	Author	Country	Age (years)	Sex	Clinical symptoms	Physical examination	Elevated tumor markers	CT findings	Location	Methods of obtaining specimens	Biopsy report	Follow-up (months)	recurrence
1	Martin et al. (4)	USA	50	M	Abdominal pain	Abdominal mass	None	CM	Intrahepatic duct (L)	Surgery	Dilated bile ducts lined by papillary projections.	6	No
			46	M	Abdominal pain	unremarkable	None	CM, IHD	Intrahepatic duct (L)	Surgery		30	Yes
			38	M	Jaundice	Jaundice	None	IHD	Intrahepatic duct (R)	Surgery		8	No
2	Terada et al. (5)	Japan	63	M	Abdominal pain and jaundice	Jaundice	CA19-9, CEA	UCM	Intrahepatic duct (L)	Percutaneous drainage	Cells are oncocytic and in a papillary fashion.	30	No
3	Nakanishi et al. (6)	Japan	63	M	Abdominal pain and jaundice	Jaundice	None	Nodular lesion	Hilar duct	Choledochoscopy	Cells show eosinophilic cytoplasm and prominent nucleoli.	75	No
4	Tanaka et al. (7)	Japan	59	F	None	unremarkable	None	PCM	Intrahepatic duct	Bile cytology was negative in 2 cases and detected atypical cells in 1 case; surgery	Papillary structures with fibrovascular cores. Cells have eosinophilic granular cytoplasm.	10	No
			58	F	Abdominal pain		None	PCM	Intrahepatic duct (L)			28	No
			62	F	Abdominal pressure		None	UCM	Intrahepatic duct (L)			112	No
			51	F	None		CA19-9	PCM	Intrahepatic duct (R)			6	Yes
			68	F	Abdominal symptoms		None	Cholangiectasis	Hilar duct			60	No
			64	M	None		None	Cholangiectasis	Hilar duct			19	No
5	Liszka et al. (8)	Poland	71	M	None	ND	None	ND	Common bile duct	Surgery	IOPN	ND	ND
6	Cocieru et al. (9)	USA	39	M	Abdominal pain	ND	None	CM	Intrahepatic duct (L)	Brushing was negative; surgery	Cells show mild to moderate atypia and oncocytic cytoplasm.	36	No
7	Kato et al. (10)	Japan	66	M	Right back pain	unremarkable	None	Cholangiectasis	Middle bile duct	Surgery	Cells are eosinophilic and papillary in shape.	51	No
8	Kakisaka et al. (11)	Japan	65	M	Epigastric pain	ND	None	CM	Intrahepatic duct (R)	Surgery	Cells have eosinophilic cytoplasm and present as papillary growths.	12	No
9	Watanabe et al. (12)	Japan	59	M	Abdominal fullness	ND	None	CM	Intrahepatic duct (R)	Surgery	Atypical papillary epithelium	40	No
10	Jurczyk et al. (13)	USA	51	M	Recurrent cholangitis	ND	ND	ND	Intrahepatic duct (L)	Ultrasound-guided percutaneous FNA	Findings consistent with IOPN, intermediate grade.	19	No
11	Tong et al. (14)	USA	61	M	Abdominal pain	ND	ND	CM	Intrahepatic duct (R)	Surgery	Findings are compatible with IOPN.	12	No
12	Tsujimae et al. (15)	Japan	66	M	None	Tenderness in epigastrium	None	PCM	Intrahepatic duct (R)	Surgery	Findings consistent with IPNB.	12	No
13	Liu et al. (16)	China	44	F	None	unremarkable	ND	CM	Intrahepatic duct (L)	Surgery	IOPN arising in bile duct	18	No

CM, cystic mass; F, female; FNA, fine-needle aspiration; IHD, intrahepatic ductal dilation; L, left; M, male; ND, no description; PCM, polycystic mass; R, right; the USA, the United States; UCM, unilocular cystic mass.

Among these patients, 15 (75%) had normal tumor markers. However, two (10%) patients had elevated CA19-9, and one (5%) had elevated CEA. Fifteen (75%) patients had intrahepatic bile duct tumors, three (15%) had hilar duct tumors, and two (10%) had common bile duct tumors. CT of the intrahepatic bile duct tumors mainly showed single or multiple cystic masses with or without bile duct dilation, and the cysts were directly connected to the bile ducts. The main CT findings of extrahepatic bile duct tumors were nodular lesions within the dilated bile duct. Similar to these CT findings, magnetic resonance imaging (MRI) of the intrahepatic bile duct tumors typically showed single or multiple cystic lesions of varying sizes. Moreover, extrahepatic bile duct tumors were characterized by papillary protuberances and bile duct dilatation. These lesions appeared hypointense in T1-weighted images and hyperintense in T2-weighted images. Unfortunately, no information about MRCP findings were available, probably because most were intrahepatic bile duct tumors. Among these patients, 17 (85%) lacked pathological diagnosis before surgery. Four patients underwent cytological examination, but three had negative results, while atypical cells were found in one patient. Two (10%) of the pathological specimens were obtained by percutaneous drainage or fine-needle aspiration, and one (5%) of the tissue specimens was obtained by cholangiocarcinoma dochoscopy.

All patients received surgical treatment without adjuvant chemotherapy. The mean follow-up time was 30.7 months (minimum value: 6 months; maximum value: 112 months). Only two (10.5%) patients experienced recurrence while one (5%) lacked a full description.

### 2.3 Discussion

Few reports exist on IOPN of the bile duct. We only found 20 cases with complete data in PubMed. Importantly, only 19% of the patients had a pathological diagnosis before surgery, which may lead to a misdiagnosis or missed diagnosis. This condition is more likely to happen when patients have concomitant gallstones, as we have reported.

Previous research has suggested that IPNB is more common in older men (17). As a type of IPNB, IOPN may have similar characteristics. The mean age of the patients with IOPN of the bile duct was 57.8 years, and 66.7% of the cases were men. However, the accuracy of these figures may be affected by the small sample size. Additionally, 66.7% of the patients with IOPN of the bile duct were from Asia which may be associated with hepatolithiasis and cholelithiasis (18).

IOPN of the bile duct exhibits no typical clinical manifestations or sensitive tumor markers. Moreover, the tumor can present features similar to those of gallstones when it causes biliary obstruction and infection. CT and MRI of IOPN of the intrahepatic bile duct mainly show single or multiple cystic masses with bile duct dilatation, and the cysts were directly connected to the bile ducts. The typical appearance of other bile duct tumors is the solid mass with irregular margins, which is easier to identify (19). However, biliary cystadenoma/cystadenocarcinoma has a similar presentation to IOPN. The key difference is that the cystic masses of the former are not associated with the bile duct. Moreover, biliary cystadenoma/cystadenocarcinoma only causes the dilatation of the proximal bile duct. In contrast, IOPN can cause diffuse dilatation of intrahepatic and extrahepatic bile ducts when the flow of mucin obstructs the papilla of Vater (18, 20). CT, MRI, and MRCP of IOPN of the extrahepatic bile duct commonly display papillary protuberances within the dilated bile duct. Identifying this condition from other types of the extrahepatic bile duct tumors is difficult, and depends on pathology (21, 22).

Pathology is the gold standard for the diagnosis of IOPN of the bile duct. The main histological manifestations are complex papillary structures with fibrovascular cores (1). Tumor cells typically contain abundant eosinophilic granular cytoplasm and round, large, and uniform nuclei (3, 23, 24). In addition, the nucleolus is obvious (25) and cell atypia is light to moderate. Tumor cells can form an intraepithelial lumen, and some are sieve shaped with a large amount of mucus (17). However, only 19% of the patients with IOPN of the bile duct had pathological diagnosis before surgery because tissue specimen acquisition is extremely difficult.

Some methods are used to obtain specimens of extrahepatic bile duct tumors, such as cytology brushing and fluoroscopic biopsy by ERCP, endoscopic ultrasonography-guided biopsy, and bile cytology (22). However, the sensitivity of cytological examination in the diagnosis of biliary stricture was only 45% (26). Although four patients with IOPN of the bile duct underwent cytological examination, three had negative results while atypical cells were found in one patient. Reports indicate that the sensitivity of fluoroscopic biopsy is 50% (27). However, the use of this method in patients with IOPN of the bile duct has not been reported. Tissue specimens from intrahepatic bile duct tumors can be obtained by percutaneous biopsy. However, this method is not recommended for cystic lesions because of the risk of bile leakage and needle tract seeding (28). Although obtaining a preoperative pathological diagnosis is suggested, the acquisition of specimens is challenging.

Peroral choledochoscopy may be the solution to this problem. The third-generation SpyGlass peroral choledochoscope was launched in 2018 with the advantages of visual examination and targeted biopsies. We can directly identify the tumors and stones visually and detect precancerous lesions of the biliary mucosa. A meta-analysis showed that the specificity and sensitivity of choledochoscopic visual diagnosis were 86% and 93%, respectively (29). In our case, choledochoscopy of IOPN showed many papillary protuberances surrounded by fish-egg-like mucosa. The reason may be that the tumor cells arrange in papillary shape and the surrounding mucosa presents intraepithelial micropapillary or flat neoplastic lesion (30). Their grades and subtypes are similar or identical to the main tumor (17). Therefore, the surrounding mucosal cells also contain a large amount of eosinophilic cytoplasm and mucin, which makes the cells swollen and shaped like fish eggs. The choledochoscopic features of this disease have not been previously reported. Additionally, we can perform the biopsy under the direct vision of choledochoscope and it has a higher sensitivity than that of fluoroscopic biopsy for the diagnosis of biliary stricture (31, 32). Choledochoscopy can also be used to treat difficult bile duct stones (33) and perform radiofrequency ablation of bile duct tumors (34).

Complete resection is currently considered the most effective treatment for IOPN of the bile duct. Tumor metastasis to other organs or systems was not detected in any of the 21 patients and they all underwent surgical treatment without adjuvant chemotherapy. Only 10% of the patients had recurrence (the mean follow-up time was 29.5 months). Therefore, the prognosis of the disease appears excellent, but this assertion needs to be confirmed by additional studies.

In conclusion, IOPN of the bile duct is extremely rare. The main clinical features are summarized based on only a few cases. Therefore, many aspects need to be further studied and confirmed. Imaging examination may lead to misdiagnosis or missed diagnosis, especially when the tumor is accompanied by gallstones. However, use of peroral choledochoscopy may solve this clinical problem.

## Data availability statement

The original contributions presented in the study are included in the article/supplementary material. Further inquiries can be directed to the corresponding author.

## Ethics statement

Written informed consent was obtained from the individual(s) for the publication of any potentially identifiable images or data included in this article.

## Author contributions

CX: Conceptualization, Data curation, Formal analysis, Investigation, Methodology, Writing – original draft. HZ: Data curation, Investigation, Resources, Visualization, Writing – original draft. YM: Formal analysis, Resources, Validation, Writing – review & editing. BC: Conceptualization, Funding acquisition, Supervision, Writing – review & editing.
